# Structural insights into the activity and regulation of human Josephin-2

**DOI:** 10.1016/j.yjsbx.2019.100011

**Published:** 2019-08-21

**Authors:** Kimberly C. Grasty, Stephen D. Weeks, Patrick J. Loll

**Affiliations:** Department of Biochemistry and Molecular Biology, Drexel University College of Medicine, Philadelphia, PA 19102, USA

**Keywords:** Deubiquitinating enzyme, Ubiquitin, Ataxin-3, Machado-Joseph disease, Crystallography

## Abstract

The MJD family of human deubiquitinating enzymes contains four members: Ataxin-3, the ataxin-3-like protein (AT3L), Josephin-1, and Josephin-2. All share a conserved catalytic unit known as the Josephin domain. Ataxin-3 and AT3L also contain extensive regulatory regions that modulate their functions, whereas Josephins-1 and -2 are substantially smaller, containing only the Josephin domain. To gain insight into how these minimal Josephins differ from their larger relatives, we determined the 2.3 Å X-ray crystal structure of human Josephin-2 and probed the enzyme’s substrate specificity. Several large disordered loops are seen in the structure, suggesting a highly dynamic enzyme. Josephin-2 lacks several allosteric sites found in ataxin-3, but its structure suggests potential regulation via ubiquitination of a loop adjoining the active site. The enzyme preferentially recognizes substrates containing K11, K48, and K63 linkages, pointing toward a possible role in maintenance of protein quality control.

## Highlights

• Josephins-1 and -2 are low molecular-weight members of the MJD family of deubiquitinating enzymes.
• Josephin-2 was shown to cleave K11 ubiquitin linkages, in addition to K48, K63, and mixed linkages.
• The crystal structure of human Josephin-2 was determined.
• The structure suggests a potential mechanism for enzyme regulation via mono-ubiquitination.

## Graphical abstract

**Figure:**
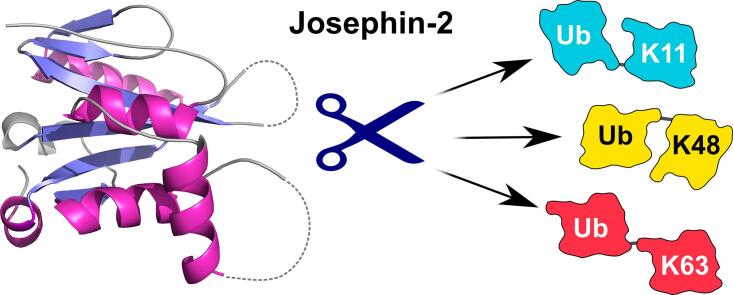

## 1 Introduction

Ubiquitination—the covalent addition of ubiquitin and ubiquitin polymers to target proteins—is a fundamental regulatory mechanism modulating both normal and pathological cellular processes (Gilberto and Peter, 2017, Lin and Machner, 2017, Uckelmann and Sixma, 2017, Varshavsky, 2017). Ubiquitination is reversible, and ubiquitin addition and removal exist in dynamic balance. Ubiquitin addition is driven by many different enzymes and regulatory proteins, which control conjugation of ubiquitin to targets, modulate ubiquitin levels, direct ubiquitination machinery to various cellular destinations, and impart target specificity. Ubiquitin removal is similarly complex; in humans, this process is catalyzed by more than one hundred different deubiquitinating enzymes (DUBs), which vary widely in their specificity, regulation, and cellular localization (Coyne and Wing, 2016, Leznicki and Kulathu, 2017, Ronau et al., 2016). DUBs can be classified into six distinct families, the smallest of which is the MJD family (Mevissen and Komander, 2017).

Enzymes of the MJD family are cysteine proteases; they are widely distributed throughout eukaryotes, being found in every phylogenetic lineage except for the excavates (Hutchins et al., 2013). Four MJD-family enzymes are present in humans: Ataxin-3, the ataxin-3-like protein (AT3L), Josephin-1, and Josephin-2. Ataxin-3 and AT3L each contain an N-terminal catalytic “Josephin” domain of approximately 180 amino acids, followed by an intrinsically disordered region of about the same size (Masino et al., 2003, Sicorello et al., 2018). These C-terminal intrinsically-disordered regions contain nuclear localization sequences, ubiquitin-interacting motifs, and polyglutamine tracts (Berke et al., 2005, Kawaguchi et al., 1994, Macedo-Ribeiro et al., 2009). Josephin-1 and Josephin-2 lack these extended C-terminal regions, and thus consist of only the Josephin domain. AT3L is thought to have arisen from ataxin-3 via a recent gene duplication in primates, whereas Josephin-1 and Josephin-2 are considered “ohnologs,” close relatives generated during whole-genome duplication events that occurred early in vertebrate evolution (Ohno et al., 1968, Vlasschaert et al., 2017).

The MJD family name is derived from Machado-Joseph disease, a neurologic disorder caused by expansion of the polyglutamine tract in ataxin-3 (Costa Mdo and Paulson, 2012). This connection to human disease has made ataxin-3 the best-studied member of the MJD family. Outside of the disease context, ataxin-3 acts as a cytoprotective agent by supporting protein quality-control pathways and DNA repair (Matos et al., 2019). The first 3-D structure for any MJD-family protein was obtained for the Josephin domain of ataxin-3 (Nicastro et al., 2005), and structures are now also available for the Josephin domain in complex with ubiquitin (Nicastro et al., 2010, Sanfelice et al., 2014, Satoh et al., 2014). AT3L is less well-studied than ataxin-3, but has been shown to deubiquitinate the transcriptional regulator Krüppel-like factor 5, thereby potentially contributing to breast cancer progression (Ge et al., 2015). The structure of the AT3L Josephin domain is also known, and is highly similar to that of ataxin-3 (Weeks et al., 2011). Josephin-1 and Josephin-2 have received less attention than ataxin-3 and AT3L, and prior to this work, no structural information was available for either Josephin-1 or Josephin-2. Josephin-1 contributes to the regulation of membrane dynamics and endocytosis (Seki et al., 2013); in contrast, relatively little is known about the biological functions of Josephin-2, although the enzyme is highly conserved in mammals, and is expressed in a broad variety of tissues, including the central nervous system (Papatheodorou et al., 2018).

To gain insight into the function and potential biological roles of Josephin-2, we determined the X-ray crystal structure of the enzyme in complex with ubiquitin at 2.3 Å resolution. The enzyme adopts a Josephin-domain fold, but nonetheless departs significantly from the structures of ataxin-3 and AT3L. Functional analyses demonstrate that Josephin-2 preferentially cleaves K11-, K48-, and K63-linked ubiquitin conjugates.

## 2 Results and discussion

### 2.1 Structure determination

In order to form a Josephin-2-ubiquitin complex sufficiently stable for crystallization, we covalently linked the enzyme to its substrate using ubiquitin_1–75_-chloroethylamine, an active site-directed ubiquitin analog (Ovaa et al., 2005). The structure of the complex was determined by multi-wavelength anomalous dispersion (MAD) phasing methods, combining data from a crystal soaked with the mercury derivative thimerosal and a crystal of selenomethionine-substituted protein. The crystal asymmetric unit contains a single copy of the ubiquitin-Josephin-2 complex. The final model contains residues 12 through 186 of Josephin-2 and the entire ubiquitin molecule. Three significant stretches of disorder are present, suggesting a highly dynamic enzyme; the disordered regions correspond to amino acids 1–11, 52–64, and 112–121. Details of the structure determination and refinement are given in Table 1.

**Table 1:** Data collection and refinement statistics.

Data Collection Statistics	*SeMet, peak*	*SeMet, inflection*	*SeMet, remote*	*Thimerosal, peak*	*Thimerosal, inflection*	*Thimerosal, remote*
Diffraction source	Beamline X-6A, NSLS	Beamline X-6A, NSLS	Beamline X-6A, NSLS	Beamline X-6A, NSLS	Beamline X-6A, NSLS	Beamline X-6A, NSLS
Wavelength (Å)	0.9787	0.9792	0.9000	1.0053	1.0089	0.9700
Temperature (K)	100	100	100	100	100	100
Detector	ADSC Quantum 210	ADSC Quantum 210	ADSC Quantum 210	ADSC Quantum 210	ADSC Quantum 210	ADSC Quantum 210
Resolution range (Å)^a^	20.0–2.30 (2.38–2.30)	20.0–2.30 (2.38–2.30)	20.0–2.30 (2.38–2.30)	20.0–2.40 (2.49–2.40)	20.0–2.40 (2.49–2.40)	20.0–2.40 (2.49–2.40)
Spacegroup	*P*6_1_22	*P*6_1_22	*P*6_1_22	*P*6_1_22	*P*6_1_22	*P*6_1_22
Unit cell						
*a, b, c* (Å)	102.1, 102.1, 92.2	102.1, 102.1, 92.2	102.1, 102.1, 92.2	102.5, 102.5, 92.3	102.5, 102.5, 92.3	102.5, 102.5, 92.3
α, β, γ (°)	90.0, 90.0, 120.0	90.0, 90.0, 120.0	90.0, 90.0, 120.0	90.0, 90.0, 120.0	90.0, 90.0, 120.0	90.0, 90.0, 120.0
Total number of observations	991,381 (57,210)	501,580 (28,454)	550,678 (52,246)	776,078 (36,542)	394,520 (17,590)	423,798 (23,240)
Number of unique reflections	12,866 (1237)	13,002 (1236)	13,070 (1273)	11,261 (1089)	11,552 (1073)	11,607 (1129)
Average multiplicity	77.0 (46.2)	38.6 (23.0)	42.1 (41.0)	68.9 (33.6)	34.1 (16.4)	36.5 (20.6)
Completeness (%)	98.3 (97.4)	99.4 (97.3)	99.7 (99.7)	96.5 (96.0)	98.9 (95.0)	99.4 (100)
Mean I/sigma(I)	81.6 (5.3)	58.0 (3.8)	53.8 (4.6)	86.2 (7.0)	62.1 (4.9)	59.5 (4.9)
Estimated Wilson B-factor (Å^2^)	50.4	50.2	49.8	50.3	50.3	50.4
R-merge^b^	0.058 (0.990)	0.054 (0.954)	0.065 (1.091)	0.054 (0.582)	0.050 (0.570)	0.058 (0.648)
R-meas^c^	0.058 (1.00)	0.055 (0.975)	0.066 (1.105)	0.055 (0.591)	0.050 (0.588)	0.059 (0.665)
R-pim^d^	0.007 (0.145)	0.009 (0.201)	0.010 (0.171)	0.006 (0.100)	0.008 (0.142)	0.010 (0.144)
CC_1/2_^e^	1.000 (0.949)	1.000 (0.911)	1.000 (0.945)	1.000 (0.971)	1.000 (0.934)	1.000 (0.937)
CC_anom_^f^	0.912 (0.053)	0.591 (0.034)	0.648 (−0.002)	0.942 (0.030)	0.754 (0.035)	0.843 (0.015)

Refinement and Model Statistics						
Resolution range (Å)^a^	19.82–2.30 (2.38–2.30)					
Number of reflections used^g^	23,358 (2209)					
Reflections used for R-free^g^	1134 (1 2 1)					
Rwork	0.201 (0.279)					
Rfree	0.224 (0.341)					
Solvent content (%)	48.0					

Number of non-hydrogen atoms						
Protein	1805					
Solvent	24					
Average B-value (Å^2^)	72.0					
RMS deviations from ideality						
Bonds (Å)	0.002					
Angles (°)	0.50					

Residue distribution in Ramachandran plot						
Most favored region (%)	99.1					
Allowed (%)	0.9					
Outliers (%)	0.0					
Clashscore	1.92					

a Values in parentheses refer to the highest resolution shell.

b *R*_merge_ is calculated by the equation *R_merge_ =* Σ*_hkl_* Σ*_i_ |I_i_*(*hkl*) − 〈*I*(*hkl*)〉*|*/Σ*_hkl_* Σ*_i_ I_i_*(*hkl*), where *I_i_*(*hkl*) is the *i*th measurement.

c *R*_meas_ (or redundancy-independent *R*_merge_) is calculated by the equation *R_meas_ =* Σ*_hkl_*[*N*/(*N −* 1)]^1/2^ Σ*_i_ |I_i_*(*hkl*) −  〈*I*(*hkl*)〉*|*/Σ*_hkl_* Σ*_i_ I_i_*(*hkl*), where *I_i_*(*hkl*) is the *i*th measurement and *N* is the redundancy of each unique reflection *hkl*.^60^

d *R*_pim_ is calculated by the equation *R*_pim_ *=* Σ*_hkl_*[1/(*N −* 1)]^1/2^ Σ*_i_ |I_i_*(*hkl*) − 〈*I*(*hkl*)〉*|*/Σ*_hkl_* Σ*_i_ I_i_*(*hkl*), where *I_i_*(*hkl*) is the *i*th measurement and *N* is the redundancy of each unique reflection *hkl*.^61^

e *CC*_1/2_ is the correlation coefficient between two randomly chosen half data sets.^62^

f *CC*_anom_ is the *CC*_1/2_ value calculated for anomalous data.

g F(+) and F(−) were treated as distinct reflections during refinement.

### 2.2 Overview of the structure

**Fig. 1:**
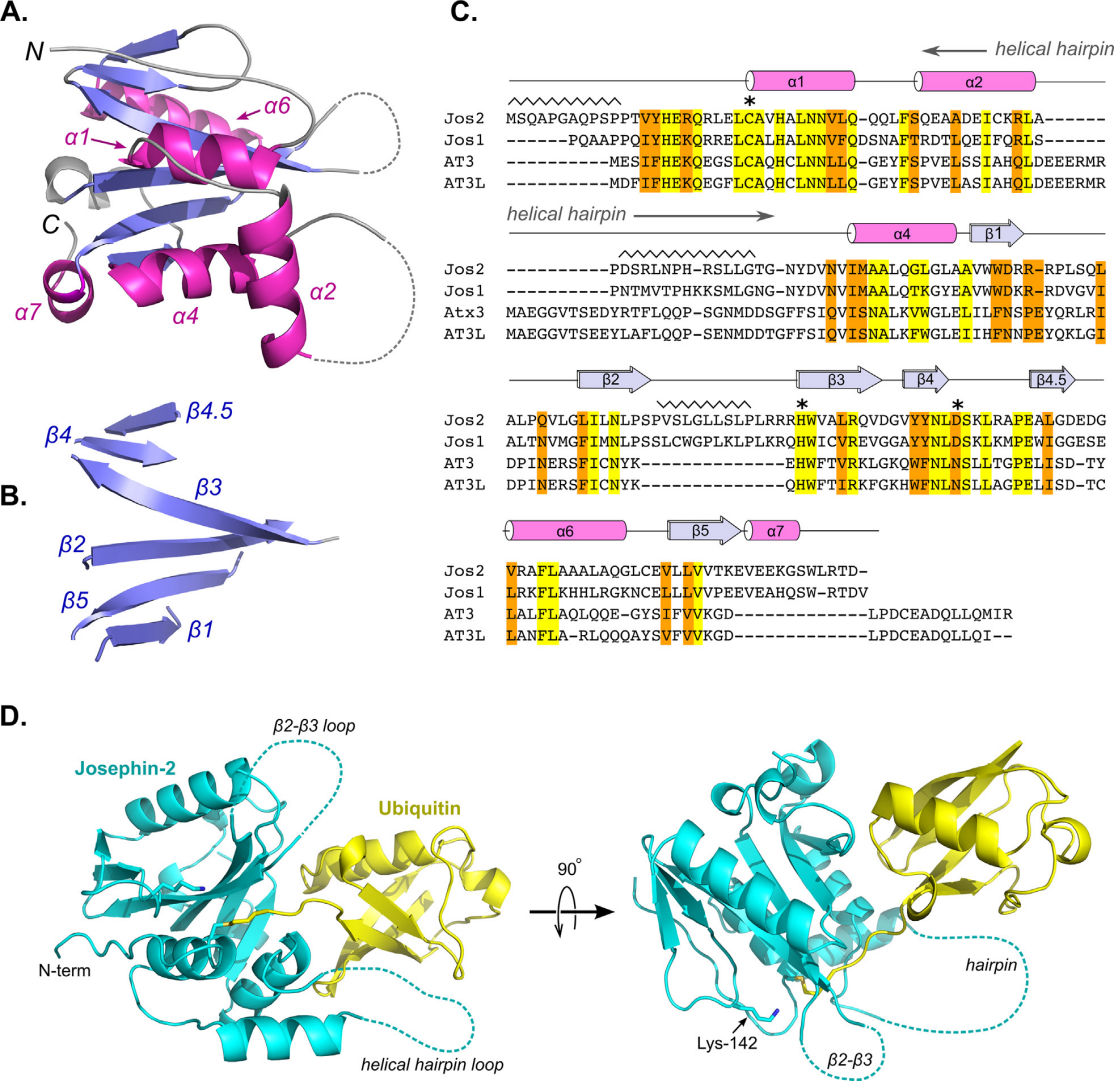
Human Josephin-2 adopts a compact α/β/α sandwich fold. (A) Cartoon representation of the Josephin-2 structure, with helices colored magenta and strands colored blue. A stereo version of this panel can be found in Fig. S1. (B) The same view as in panel A, but with the helices removed to reveal the central sheet. (C) Sequence alignment of the four members of the human MJD family. *Abbreviations used:* Jos2, Josephin-2; Jos1, Josephin-1, AT3, ataxin-3. Identities are colored yellow, and similar residues are colored orange. The secondary structure breakdown for Josephin-2 is shown above the sequence. Regions with the sawtooth symbol represent disordered stretches in the crystal structure, and the catalytic triad is marked by asterisks. The position of the helical hairpin in ataxin-3 and AT3L is also indicated. The alignment was prepared using TM-align and EMBOSS-Needle (Madeira et al., 2019, Zhang and Skolnick, 2005). (D) Two views of the complex between Josephin-2 (cyan) and ubiquitin (yellow). Large disordered loops in Josephin-2 are shown as dashed lines. The position of Lys-142 in the β4-β4.5 loop is shown in the right-hand panel. (For interpretation of the references to colour in this figure legend, the reader is referred to the web version of this article.)

The Josephin-2 molecule assumes a compact α/β/α sandwich structure, containing a central six-stranded beta sheet with helices packed against either face (Fig. 1). The secondary structural elements are numbered according to the same scheme that is used for ataxin-3 and AT3L. One face of the central sheet is covered by helices 1 and 4, as well as an extended portion of the N-terminus corresponding to residues 12–19. The opposite face of the sheet is covered by helices 6 and 7 and an irregular loop connecting beta strands 1 and 2. A 28-residue loop protrudes from the enzyme’s compact α/βα core, occupying a position analogous to that of the α2–α3 helical hairpins in the ataxin-3 and AT3L structures (Nicastro et al., 2005, Weeks et al., 2011). In Josephin-2, most of this loop is disordered, apart from two and a half turns of helix 2 at the beginning of the loop; the region that would correspond to helix 3 is not observed in the Josephin-2 structure. Additionally, the last strand on the edge of the beta sheet has no equivalent in the ataxin-3 or AT3L structures. Because this short strand falls between beta strands 4 and 5 in the sequence, we have labeled it strand 4.5 (Fig. 1).

**Fig. 2:**
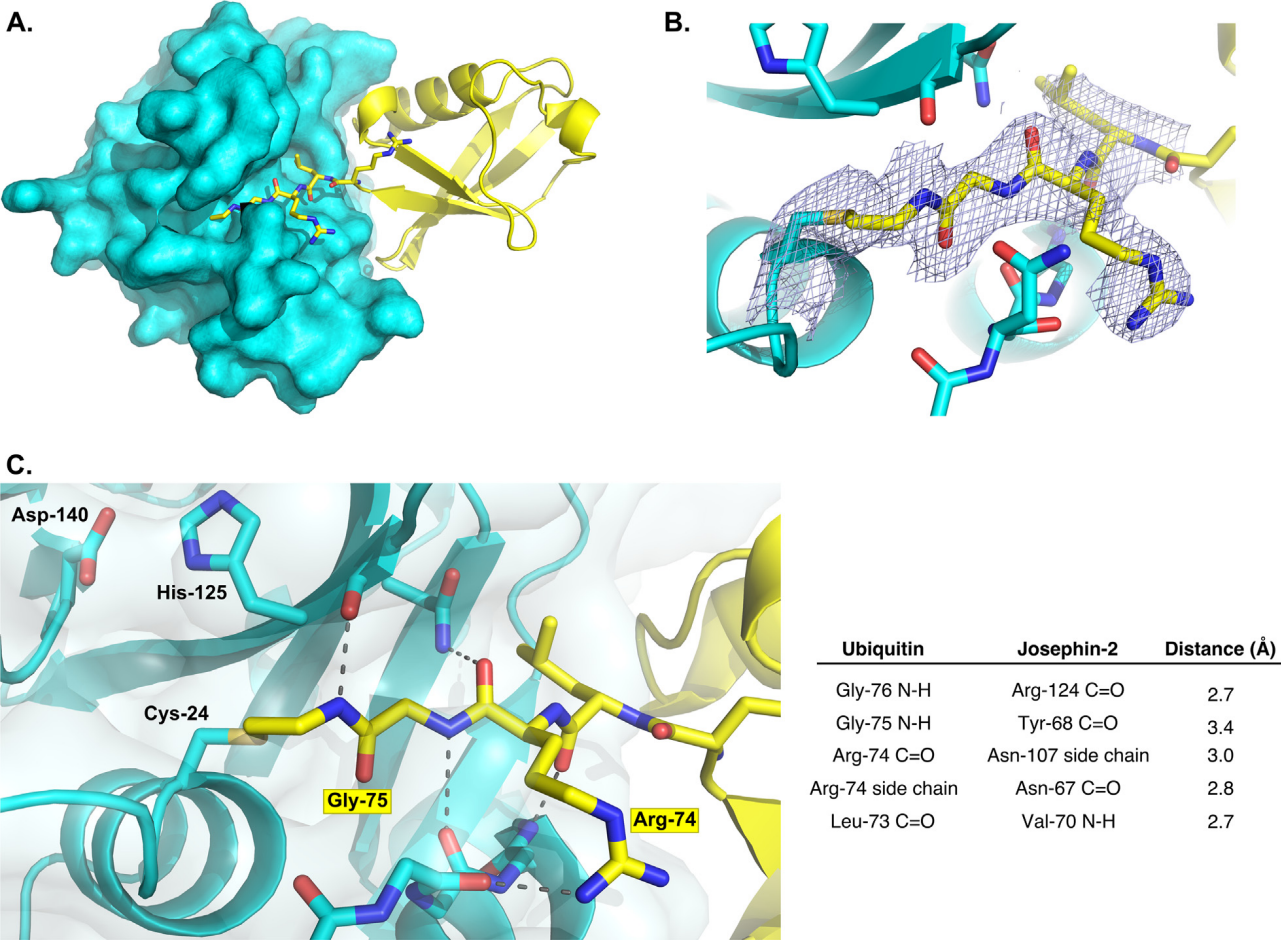
Details of the Josephin-2 active site. (A) The extended C-terminus of ubiquitin (yellow) threads into the active site of Josephin-2 (shown as a cyan surface representation). (B) A portion of the final 2Fo-Fc map, contoured at 1σ, showing the site of covalent attachment of the ubiquitin molecule to Cys-24. (C) Detailed view showing polar interactions between the ubiquitin C-terminus and active-site Josephin-2 residues. Hydrogen bonds shown as dashed lines are listed in the table at right. The Cys-Asp-His residues of the Josephin-2 catalytic triad are labeled. A stereo version of this panel can be found in Fig. S1. (For interpretation of the references to colour in this figure legend, the reader is referred to the web version of this article.)

The ubiquitin molecule is packed against the side of Josephin-2’s α/β, sandwich, nestled in a cleft between the core of the enzyme and helix 2; this position corresponds to the site on ataxin-3 identified by Nicastro et al. as Site 1 (Nicastro et al., 2010). The extended ubiquitin C-terminus is threaded into the Josephin-2 active site (Fig. 2), and thus the ubiquitin’s position corresponds to that of the distal molecule during cleavage of a ubiquitin-ubiquitin isopeptide bond. A number of interactions within the enzyme’s active site serve to position the ubiquitin C-terminus (described below). Apart from these, the only other contacts between ubiquitin and Josephin-2 are three hydrogen bonds: One links the side chain of Josephin-2’s Gln-78 with the backbone carbonyl oxygen of ubiquitin Thr-9, while two water-mediated hydrogen bonds connect the carbonyl oxygen of ubiquitin Glu-34 with the backbone amide and carbonyl atoms of Josephin-2 Trp-86.

**Fig. 3:**
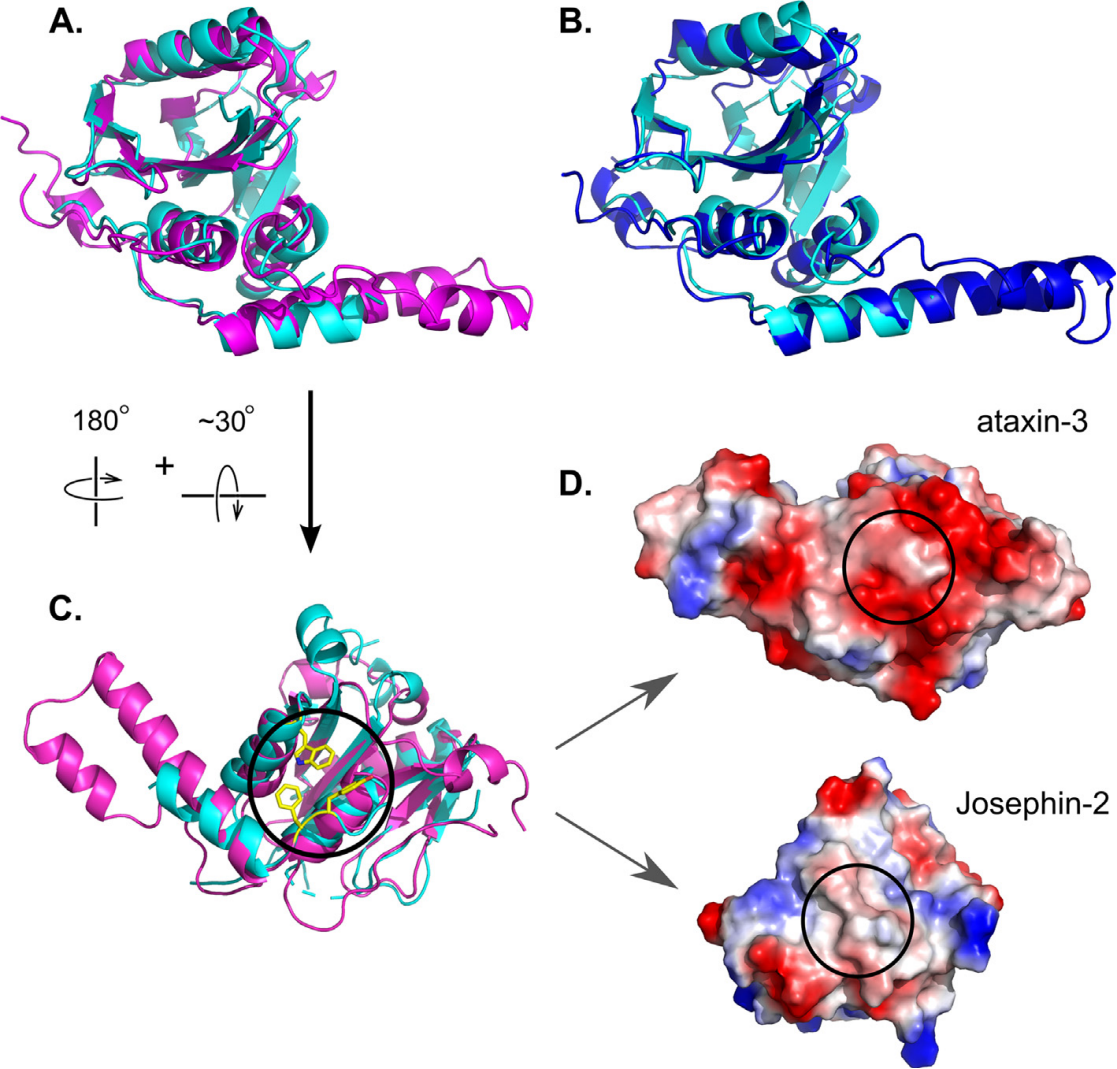
The overall Josephin fold is conserved between members of the MJD family. Josephin-2 (cyan) is shown superposed on ataxin-3 (magenta; panel A) and AT3L (blue; panel B). (C) A reversed view of the superposed Josephin-2 and ataxin-3 structures, showing the “rear” surface of the molecules, i.e. the opposite face from the surface containing the active site. The black circle indicates Site 2 of ataxin-3, with ataxin-3’s Tyr-27, Phe-28, and Trp-87 residues shown as yellow sticks. (D) Electrostatic surface representations of ataxin-3 and Josephin-2, shown in the same orientation as panel C. The black circles show the position of Site 2 in ataxin-3, and of the corresponding region in Josephin-2, highlighting how the surface characters of the two proteins differ at this position. (For interpretation of the references to colour in this figure legend, the reader is referred to the web version of this article.)

At the Josephin-2 N-terminus, residues 12–19 adopt an elongated conformation and pack against the central beta sheet. In ataxin-3 and AT3L, this elongated stretch then reverses direction in a beta turn and enters helix 1. However, in the Josephin-2 structure, no electron density was observed for this beta turn; instead, this region shows evidence for a domain swap, in which residues 12–19 from one molecule pack against the central sheet of a neighboring molecule in the crystal lattice (Fig. S2). Refinements were carried out using both the domain-swapped and non-domain-swapped structures, with the former giving marginally better statistics. However, size-exclusion chromatography experiments show no evidence for dimerization in solution (data not shown), and we therefore regard this domain swap as a crystallization artifact without physiological relevance. Apart from this domain swapping, the N-terminal residues in Josephin-2 adopt a conformation that is very similar to those seen in the N-termini of ataxin-3 and AT3L. For clarity’s sake, the non-swapped structure is shown in Fig. 1, Fig. 2, Fig. 3.

### 2.3 The Josephin-2 active site

The covalent complex between Josephin-2 and ubiquitin mimics the covalent thioester intermediate formed during the deubiquitination reaction (Ovaa et al., 2005). Clear electron density is seen for the linkage between the ubiquitin C-terminus and Cys-24 in the enzyme active site (Fig. 2). In addition to this covalent linkage, five hydrogen bonds connect the enzyme and the ubiquitin C-terminal region, and serve to stabilize and direct the substrate as it threads into the active site.

Josephin-2 contains a Cys-His-Asp catalytic triad, composed of the active-site nucleophile Cys-24, together with His-125 and Asp-140. This distinguishes the enzyme from ataxin-3, AT3L, and many other papain-like cysteine proteases, in which the triads contain asparagine rather than aspartate (Vernet et al., 1995). In Josephin-2, Asp-140 is positioned so as to accept a hydrogen bond from the Nε2 atom of His-125, meaning it could potentially serve as a general acid during the catalytic cycle, in addition to helping position the histidine. The ubiquitin molecule is covalently attached to Cys-24, which moves this residue’s side chain away from His-125 and toward the substrate-binding site. Hence, this structure represents the species formed post-nucleophilic attack, rather than the pre-catalysis species, in which Cys-24 would be expected to interact with His-125.

### 2.4 Comparison with other Josephin domain-containing proteins.

The four human proteins that contain Josephin domains segregate into two groups, ataxin-3/AT3L and Josephin-1/Josephin-2 (Nijman et al., 2005). Ataxin-3 and AT3L share 85% sequence identity, while the sequences of Josephin-1 and Josephin-2 are 51% identical. Between the two groups, however, sequence identity is substantially lower; for example, Josephin-2 shares only 23.5 and 21.8% identity with ataxin-3 and AT3L, respectively. Josephin-2 adopts the same fold as ataxin-3 and AT3L (Fig. 3), and the protein backbones can be superimposed with RMS differences in Cα positions of 2.0 and 2.5 Å for Josephin-2 versus ataxin-3 and AT3L, respectively. However, given the levels of sequence divergence, it is not surprising that the Josephin-2 structure deviates in many ways from those of the other two proteins.

When the Josephin-2 sequence is aligned with those of ataxin-3 and AT3L, most regions agree well, except for three major insertions or deletions (Fig. 1). The first is an insertion of ten residues at the Josephin-2 N-terminus. However, these residues are not ordered in the Josephin-2 crystal structure, which therefore affords no structural insights about this region. However, it is worth noting that this stretch of residues contains Ser-10, which has been observed to be phosphorylated in cells (Sharma et al., 2014).

Second, Josephin-2 contains a deletion within a region that corresponds to the helix-2/helix-3 hairpin in ataxin-3 and AT3L. This hairpin protrudes from the protein’s central core and clamps the ubiquitin substrate against the main body of the enzyme. In Josephin-2, this region is 19 residues shorter than the corresponding region in ataxin-3 and AT3L. The first half of helix 2 aligns well with the corresponding helices in ataxin-3 and AT3L, but the remainder of the region is disordered and contains no helix 3 equivalent, in line with secondary-structure predictions. Hence, unlike the case with ataxin-3 and AT3L, no stabilizing contacts occur between ubiquitin and Josephin-2’s putative hairpin, suggesting that the ubiquitin molecule is not tightly clamped in place. Consistent with this idea, the average B-value for ubiquitin is higher than that for Josephin-2 (80.8 Å^2^ vs. 68.2 Å^2^). Ubiquitin itself is highly stable and tightly packed, so the most likely explanation for its elevated B-values is that it is only loosely tethered in place, and therefore is able to explore small rigid-body excursions from its equilibrium position. Given that this disordered loop adjoins the active site, it is tempting to speculate that it might participate in recognition of larger oligomeric ubiquitin substrates, which could induce a disorder-to-order transition.

Thirdly, an insertion is found in the loop connecting beta strands 2 and 3. In ataxin-3 and AT3L, these strands are connected by a tight turn, but in Josephin-2 the loop contains an additional thirteen residues, most of which are disordered. This loop is positioned over the active site, adjacent to the bound ubiquitin molecule, and thus may also play a role in engaging larger substrates. Notably, in ataxin-3 the β2–β3 loop contains Lys-117; mono-ubiquitination at this residue stimulates enzymatic activity via transient binding of the covalently-attached ubiquitin molecule in Site 1 (Faggiano et al., 2015, Todi et al., 2010). In Josephin-2, the β2–β3 loop contains no lysines, and thus an analogous post-translational modification cannot occur. However, Josephin-2 does contain a lysine in the β4–β4.5 loop, which is adjacent to the β2–β3 loop, and this residue (Lys-142) is ubiquitinated in cells (Kim et al., 2011, Wagner et al., 2011, Zhou et al., 2018). Our structure suggests that a ubiquitin conjugated to Lys-142 would be able to reach Site 1, and therefore might plausibly serve an activating function (Fig. 1). It is not yet known whether Josephin-2 activity can be stimulated by ubiquitination; however, its close relative Josephin-1 is known to be activated by mono-ubiquitination (Seki et al., 2013). Like Josephin-2, Josephin-1 contains no lysines in the β2–β3 loop, but does have a ubiquitination site in the region corresponding to the β4–β4.5 loop (Kim et al., 2011, Zhou et al., 2018). Taken together, these observations are consistent with the possibility that ubiquitination in the Josephin-1/Josephin-2 β4–β4.5 loop activates the enzyme, analogous to ubiquitination in ataxin-3’s β2–β3 loop.

In addition to the insertions and deletions described above, Josephin-2 differs significantly from ataxin-3 and AT3L at several other positions. One is at the C-terminus, where the final approximately ten residues of Josephin-2 diverge both in sequence and structure from the analogous regions in the other two proteins. In all three proteins, the C-terminal residues form a small helix (helix 7); however, just upstream of this helix, the polypeptide chain turns in opposite directions in Josephin-2 versus ataxin-3 and AT3L. Also, in the latter two proteins, a short insertion of approximately five residues separates the terminal helix from beta strand 5, whereas in Josephin-2 the helix immediately follows the strand. Thus, in ataxin-3 and AT3L the C-terminal helix ends up near the N-terminus, while in Josephin-2 this helix sits at the edge of the central sheet, above the ubiquitin-binding site and next to the extended β1–β2 loop. There is no discernable sequence homology between Josephin-2 and the other proteins in the β1–β2 loop region; indeed, this loop is where the Josephin-2 structure deviates most from those of ataxin-3 and AT3L (Fig. S3). Thus, for this portion of the protein’s surface—on the opposite face of the molecule from the active site, where helix 7 and the β1–β2 loop cover the central sheet—Josephin-2 differs appreciably from either ataxin-3 or AT3L. There are no crystal contacts near this region of the Josephin-2 surface, and so the conformation observed likely reflects the undistorted solution conformation. However, because Josephin-2 appears to be a particularly flexible molecule, we cannot rule out that small crystallization-induced perturbations exist at other sites.

Adjacent to this region is a small surface patch that is also different in Josephin-2 as compared to ataxin-3 and AT3L; this area lies on the bottom of the molecule in the view shown in Fig. 3A. In ataxin-3, this surface patch is a second ubiquitin-binding site known as “Site 2,” and contains three key residues, Tyr-27, Phe-28, and Trp-87 (Nicastro et al., 2010, Nicastro et al., 2009). In Josephin-2, only one of these three residues is conserved (the equivalent amino acids are Leu-37, Phe-38, and Leu-80). Further, the enzyme’s surface character in this region differs substantially between ataxin-3 and Josephin-2; the surface of ataxin-3 is strongly acidic around Site 2, whereas the corresponding surface in Josephin-2 is relatively neutral (Fig. 3D). These structural differences suggest that a ubiquitin-binding site analogous to Site 2 does not exist in Josephin-2.

### 2.5 Deubiquitinating activity.

Josephin-2 cleaves ubiquitin conjugates that contain small C-terminal adducts, including the fluorogenic substrate ubiquitin-AMC and the hexahistidine-tagged species Ub-His_6_ (Orcutt et al., 2012, Seki et al., 2013, Tzvetkov and Breuer, 2007, Weeks et al., 2011). We first tested whether Josephin-2 activity toward ubiquitin-AMC could be stimulated by free ubiquitin, given that such a stimulation has been observed with ataxin-3 (Faggiano et al., 2015). However, no increase in Josephin-2 activity was observed using either monomeric ubiquitin or K48-linked di-ubiquitin, at concentrations up to 25 µM; in contrast, these conditions give marked stimulation with ataxin-3 (data not shown).

**Fig. 4:**
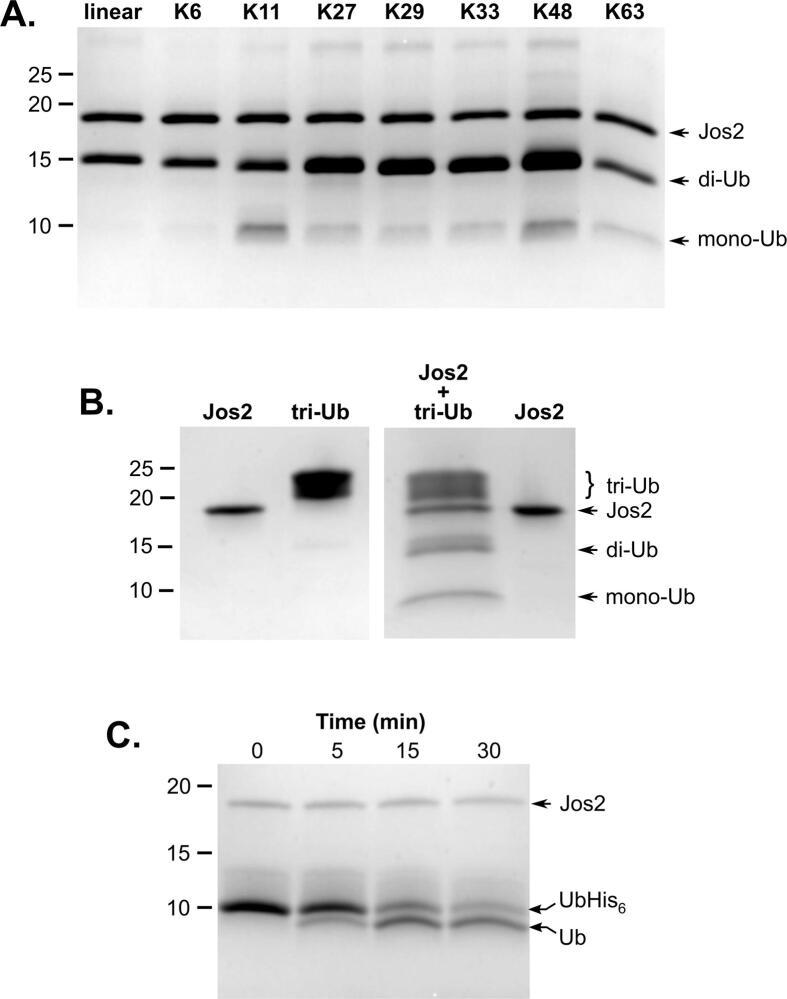
Activity of human Josephin-2 against different ubiquitin linkage types. (A) Activity of Josephin-2 versus a panel of ubiquitin dimers containing all naturally-occurring linkages. (B) Josephin-2 is able to cleave a branched K11/K48 tri-ubiquitin chain. Note that the ubiquitin trimer runs as a smeared band in this gel system. Panels A & B both show the results of a 20-hour incubation at 37°. (C) Josephin-2 cleaves the small Ub-His_6_ substrate more efficiently than ubiquitin dimers. A representative time course is shown for cleavage at room temperature.

Josephin-2 can also cleave both K63-linked and K48-linked polyubiquitin, and degrades the former more efficiently than the latter (Seki et al., 2013, Weeks et al., 2011). To gain additional insights into the substrate specificity of Josephin-2, we tested enzyme activity using a panel of di-ubiquitin conjugates representing all possible native linkage types (linear, K6, K11, K27, K29, K33, K48, and K63). Josephin-2 exhibited clear preferences for certain linkage types, with K11- and K48-linked dimers being cleaved most efficiently, and essentially no cleavage being seen for linear and K6-linked dimers (Fig. 4). Even for those dimers that are most efficiently targeted, cleavage of di-ubiquitin substrates is relatively slow, as compared to cleavage of either poly-ubiquitin or Ub-His_6_; this is similar to the behavior of ataxin-3 and AT3L (Weeks et al., 2011).

The ubiquitin linkage types most readily cleaved by Josephin-2—K11, K48, and K63—are all abundant in cells (Swatek and Komander, 2016, Xu et al., 2009). However, they are dissimilar in structure, with K11 and K48 homopolymers forming two distinct types of compact structures (Castaneda et al., 2013, Eddins et al., 2007), while K63-linked chains are more extended (Datta et al., 2009, Komander et al., 2009, Weeks et al., 2009). Therefore, Josephin-2 possesses the ability to recognize and cleave substrates with differing topologies. It is not unique in this ability; multiple instances exist of DUBs that can cleave K11, K48, and K63 linkages, with USP9X and SdeA being two recent examples (Paudel et al., 2019, Puvar et al., 2019). All three of these linkage types can be combined in mixed ubiquitin chains that efficiently drive proteasomal degradation (Grice et al., 2015, Meyer and Rape, 2014). We therefore tested whether Josephin-2 could cleave a branched K11/K48 tri-ubiquitin chain, and observed that it can indeed do so (Fig. 4).

Thus, the three types of ubiquitin linkages recognized by Josephin-2 are all involved in protein degradative pathways, and either direct proteins to the proteasome or mediate autophagy (Kwon and Ciechanover, 2017). Hence, we speculate that Josephin-2’s biological role is likely related to maintaining cellular protein quality control. *In vitro*, the enzyme cleaves small model substrates (e.g., di-ubiquitin) slowly, suggesting that Josephin-2 requires activation to accomplish its cellular functions; this may require an as-yet undiscovered partner, or involve mono-ubiquitination, as seen for other MJD-family enzymes.

## 3 Materials and methods

### 3.1 Materials

The di-ubiquitin panel was purchased from UbiQ Bio (Amsterdam, The Netherlands), and branched K11/K48 tri-ubiquitin was obtained from Boston Biochem (Boston, USA). Ub-His_6_ was prepared as described (Weeks et al., 2011).

### 3.2 Subcloning and protein expression

The human *JosD2* gene (encoding the Josephin-2 protein) was kindly provided by Dr. Randall Pittman. *JosD2* was introduced into the pETHSUL vector to encode a His_6_-SUMO-Josephin-2 construct (Weeks et al., 2007). The gene for human ubiquitin was then fused, in frame, to the 5′ end of the His-tagged SUMO gene, with the expectation that the presence of ubiquitin would promote the solubility and stability of Josephin-2. Protein was expressed at 24 °C for approximately 24 h in Rosetta2 (DE3) cells using auto-induction media (Studier, 2005). The cultures were harvested by centrifugation at 4 °C and cell pellets were stored at −80 °C. Protein isolated from over-expressing cells demonstrated an electrophoretic mobility corresponding to the expected molecular weight for the SUMO-Josephin-2 fusion, indicating that the fused ubiquitin is removed during expression, presumably by Josephin-2. Selenomethionine-labeled Josephin-2 was produced using PASM-5052 auto-induction media (Studier, 2005), and purified in the same manner as the native protein.

Cells were thawed and re-suspended in Buffer A (50 mM sodium phosphate pH 7.4, 250 mM NaCl, 7.5 mM imidazole, 10% (w/v) glycerol, 5 mM β-mercaptoethanol). Cells were lysed by three passes through an Emulsiflex cell disrupter (Avestin, Inc.), and subjected to low-speed centrifugation (27,000×*g* for 15 min), followed immediately by high-speed centrifugation (165,000×*g* for 1 h). The resulting supernatant was filtered sequentially through 5 µm and 0.45 µm syringe filters and then loaded onto a 5-mL HiTrap IMAC HP column (GE Life Sciences) at approximately 1 mL/min. The column was washed with 10–20 column volumes of Buffer A + 0.1% Triton X-100, and the protein was eluted with Buffer B (50 mM sodium phosphate pH 7.4, 250 mM NaCl, 250 mM imidazole, 10% (w/v) glycerol, 5 mM β-mercaptoethanol). Fractions corresponding to the protein peak were pooled and EDTA was added to a final concentration of 2 mM. The doubly-tagged SUMO hydrolase UD1 was then added ((Weeks et al., 2007); 0.5 mg of hydrolase/L of expression culture) and the protein was dialyzed overnight at 4 °C against two changes of Buffer A. The dialyzed sample was passed over a 5-mL HiTrap IMAC column equilibrated with Buffer A, and the UD1-cleaved protein present in the flow-through was collected and concentrated using an 10 K MWCO Amicon Ultra concentrator (Millipore). Concentrated protein was kept on ice until use.

### 3.3 Preparation of the Josephin-2-ubiquitin complex.

The ubiquitin thioester (residues 1–75) was produced and treated with 2-chloroethylamine as previously described (Weeks et al., 2011). Briefly, ubiquitin_1–75_ was expressed as a fusion with a C-terminal His_6_-tagged intein from *Mycobacterium xenopi* and purified by IMAC. Cleavage was induced by sodium mercaptoethane sulfonate (MESNA) and the His-tagged intein was removed on an IMAC column, yielding the ubiquitin C-terminal thioester. The solution pH was adjusted to 8, and 2-chloroethylamine was added, to produce the amide adduct at the ubiquitin C-terminus. The resulting activity-based probe was used to covalently attach ubiquitin to Josephin-2. Three molar equivalents of the ubiquitin probe were added to one equivalent of Josephin-2 and the proteins were incubated for 16–18 h at 16 °C. The mixture was then loaded onto a 5-mL HiTrap SP HP column (GE Life Sciences) and eluted with a salt gradient to isolate the desired complex (initial buffer = 25 mM Bicine pH 8.5, 5 mM DTT; final buffer = initial buffer + 1 M NaCl). After concentration to about 1 mL, the complex was loaded onto an S200 (16/10) size-exclusion column (GE Life Sciences) as a polishing step, with the column being equilibrated in 25 mM sodium acetate pH 4.6, 150 mM NaCl, 5 mM DTT. After overnight dialysis in 10 mM sodium acetate pH 5, 50 mM NaCl, 20% (w/v) glycerol, 5 mM DTT, the protein was concentrated to 20 mg/mL for crystallization trials using the microbatch-under-oil technique (Chayen, 1997). Diffraction-quality crystals were obtained in approximately 24 h by mixing 1 µL of the protein solution (9 mg/mL) with an equal volume of 0.1 M sodium acetate pH 4.6, 0.2 M CaCl_2_, 22.5% (w/v) PEG 6000 and incubating under Al’s Oil at 18 °C (D'Arcy et al., 1996).

### 3.4 Data collection and structure determination.

Crystals were prepared for data collection by dipping briefly in a solution of 30% (v/v) glycerol in mother liquor, followed by flash-cooling in liquid nitrogen. The mercury derivative was produced by a 10-minute soak in 10 mM thimerosal, 0.05 M sodium acetate pH 5.5, 0.2 M CaCl_2_, 15% (w/v) PEG 6000, 10 mM DMSO. A selenomethione-containing derivative was prepared using PASM-5052 media (Studier, 2005).

Three-wavelength MAD data were collected for both the thimerosal and SeMet derivatives. Data were processed using XDS (Kabsch, 2010), after which the Autosol pipeline in Phenix was used to determine phases and build an initial model (Terwilliger et al., 2009). When both the mercury and SeMet data were used in the Autosol procedure, slightly better maps were obtained than when using the mercury data alone. The SeMet data alone did not yield a solution, which is not surprising given that the Josephin-2 protein contains only two methionines, one of which (the start methionine) is disordered in the crystal. The initial model was refined against the peak data from the SeMet crystal, using alternating cycles of manual rebuilding in Coot (Emsley et al., 2010) and refinement in Phenix. Data collection and refinement statistics are shown in Table 1.

### 3.5 Ubiquitin cleavage assays

Di- and tri-ubiquitin cleavage assays were conducted at 37 °C using 3.6 µM Josephin-2 and 15 µM substrate in a buffer containing 25 mM Tris pH 7.5, 5 mM DTT**.** After 20 h, the reactions were quenched by addition of SDS sample buffer. Cleavage of the Ub-His_6_ reagent was carried out at room temperature using 12.5 µM Josephin-2 and 125 µM substrate in a buffer containing 0.1 M Tris pH 7.5. Assay solutions were analyzed by SDS-PAGE and the gels stained with Coomassie Brilliant Blue.

### 3.6 Accession numbers

Coordinates and structure factors have been deposited in the Protein Data Bank; PDB ID: 6PGV.

## CRediT authorship contribution statement

**Kimberly C. Grast:** Conceptualization, Investigation, Writing - review & editing. **Stephen D. Weeks:** Conceptualization, Investigation. **Patrick J. Loll:** Conceptualization, Investigation, Writing - original draft, Writing - review & editing, Funding acquisition.

## Declaration of Competing Interest

The authors declare that they have no known competing financial interests or personal relationships that could have appeared to influence the work reported in this paper.
