# Supplementary material for: Structural insights into the activity and regulation of human Josephin-2
Source: J Struct Biol X. 2019 Aug 21;3:100011. doi: 10.1016/j.yjsbx.2019.100011 (PMC7337049; doi:10.1016/j.yjsbx.2019.100011)
Supplement: Supplementary data 1 [file mmc1.pdf]

SUPPLEMENTARY DATA

Structural insights into the activity and regulation of human Josephin-2

Kimberly C. Grasty[a], Stephen D. Weeks[a,1], Patrick J. Loll[*a]

[a] Department of Biochemistry and Molecular Biology, Drexel University College of Medicine, Philadelphia, PA USA 19102

[1] Present address: OrthogonX, Gaston Geenslaan 1, 3000, Leuven, Belgium

[*] Corresponding author: Patrick J. Loll
Department of Biochemistry and Molecular Biology
Drexel University College of Medicine
Mailstop 497
Philadelphia, PA USA 19102
pjl28@drexel.edu
Telephone: 1 (215) 762-7706
Fax: 1 (215) 762-4452

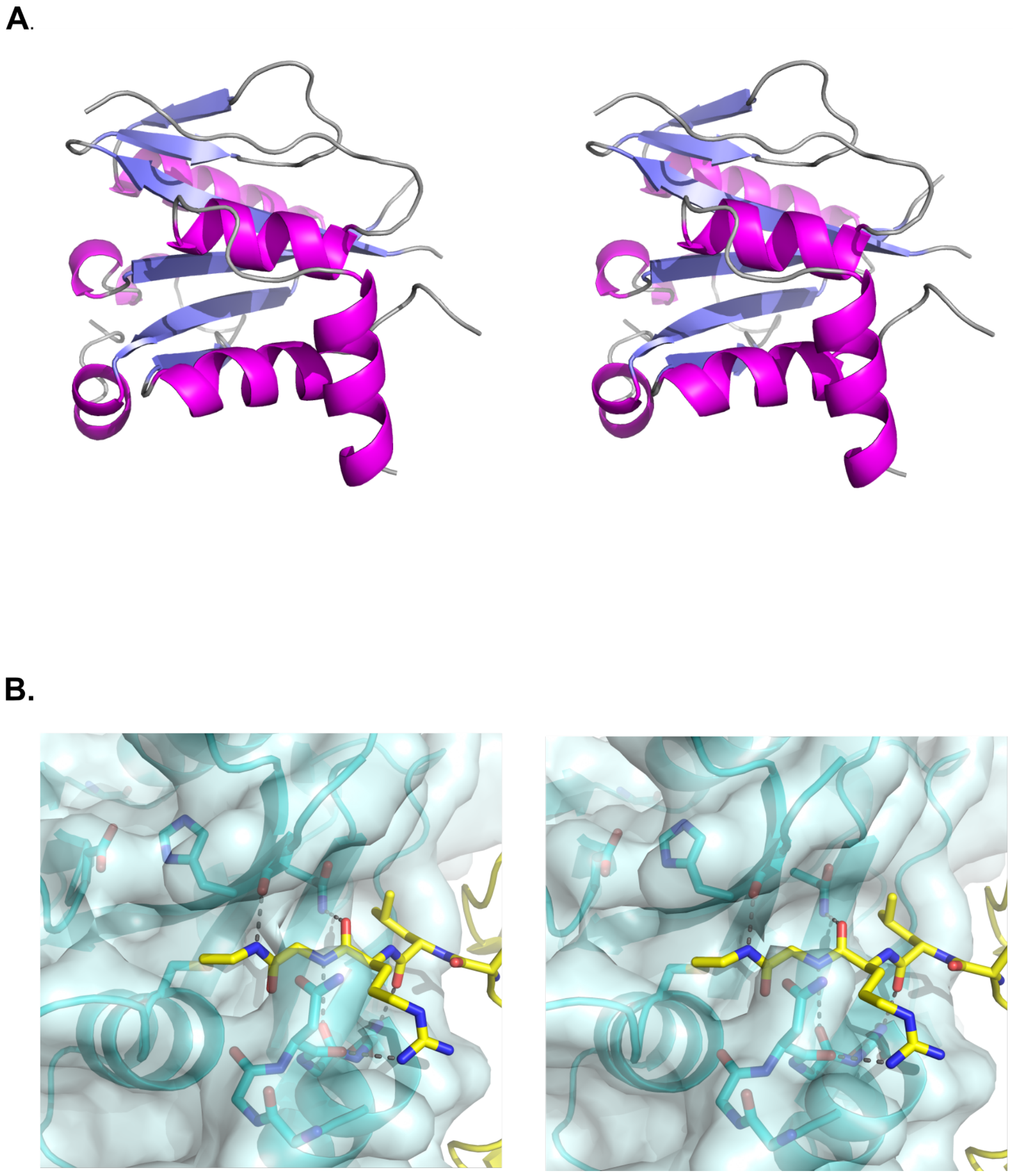

Figure S1. Divergent (wall-eyed) stereo views of the Josephin-2 structure. (A) Stereo version of Figure 1A (note that loop smoothing has been turned off in the stereo figure, leading to more complex paths for the regions of irregular secondary structure). (B) Stereo version of Figure 2C.

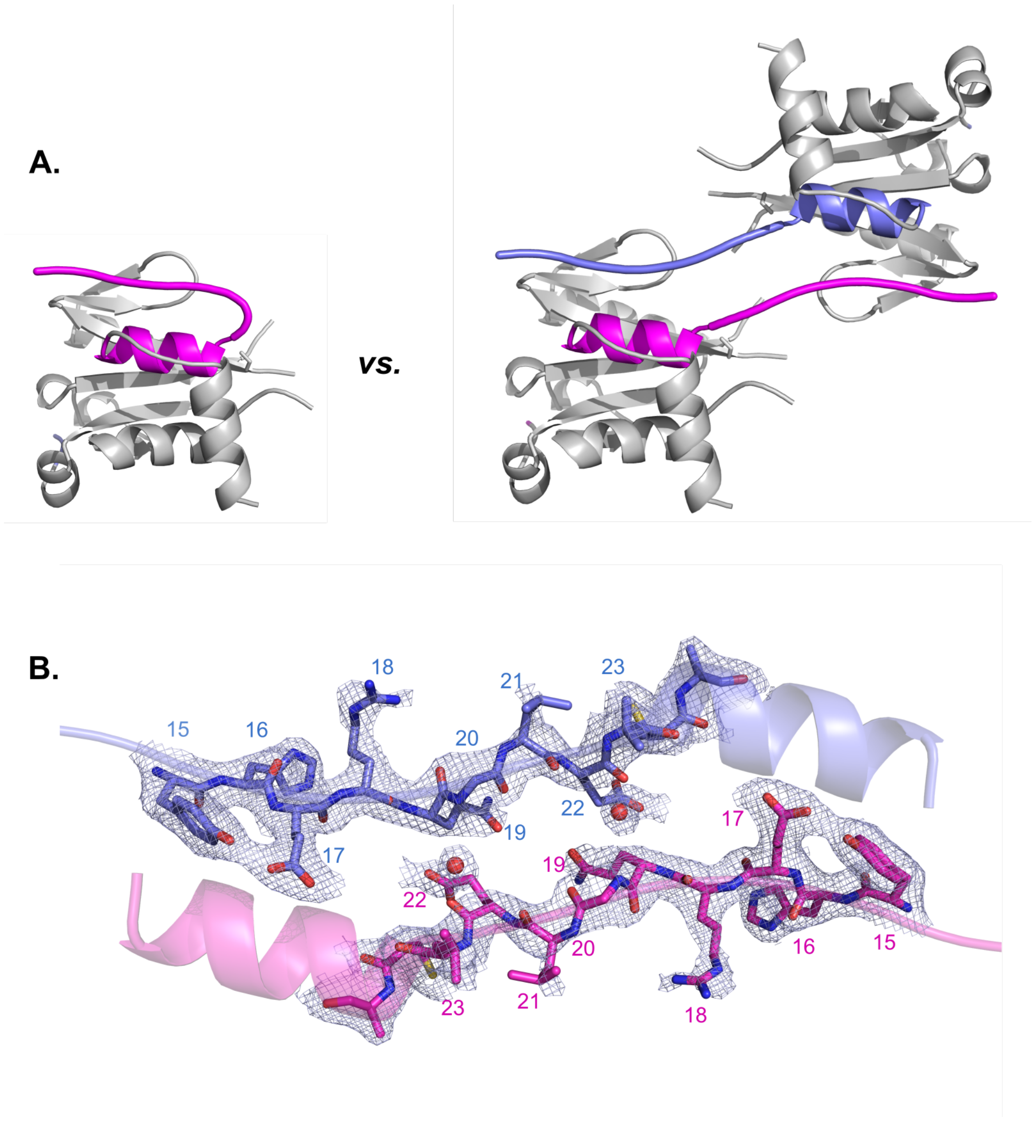

Figure S2. Domain swapping in the Josephin-2 crystal structure. (A) Schematic representation of the non-domain-swapped structure (left) versus the domain-swapped version (right). The magenta portion of the chain represents residues 12-34 in one molecule, while the blue chain represents the corresponding stretch of residues in a symmetry-related molecule. (B) An omit map calculated by omitting residues 18-22, corresponding to the point of crossover for the domain swap. This view is shown in the same orientation as panel A. The map is contoured at 0.9 σ.

A.

Josephin-2	Ataxin-3	AT3L
P12-Q34	E2-Q24	D3-Q25
Q35-A51	G25-D41	G26-D42
N67-R90	F74-P97	F75-P98
R91-P109	Y99-K117	Y100-K118
R124-D151	E118-D145	Q119-D146
D153-A160	T146-A153	T147-A154
A162-Q165	L155-E158	R155-Q158
L167-K176	G159-D168	A160-D169

B.

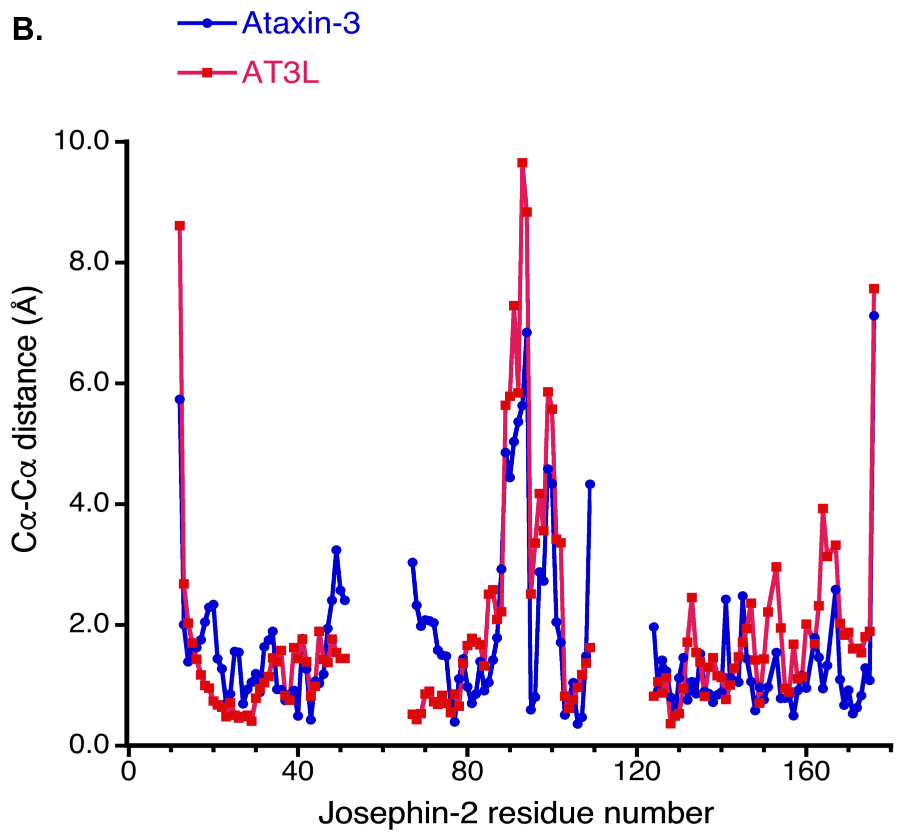

Figure S3. Differences in Cα positions after superposition: Josephin-2 vs. ataxin-3 & Josephin-2 vs. AT3L. (A) Table showing equivalent residue ranges used for superpositions. These correspond to the alignments (produced by TM-align) shown in Figure 1. A total of 132 equivalent residue pairs were included in each superposition. (B) Cα-to-Cα distance after superposition. Josephin-2 vs. ataxin-3, blue; Josephin-2 vs. AT3L, red. Superpositions were carried out using LSQKAB (Kabsch, 1976).

```
Jos2                         HHHHHHHHHH      HHHHHHHHHH                                      HHHHHHHHH   EEEEE       GGG
AT3                          HHHHHHHHH       HHHHHHHHHHHHHHHHHHHH      HHHHHHH                HHHHHHHH    EEEE
AT3L                         HHHHHHHHHH      HHHHHHHHHHHHHHHHHHH       HHHHHHH               HHHHHHHHH    EEEE   HHHH
Jos2  MSQAPGAQPSPPTVYHERQRLELCAVHALNNVLQQQLFSQEAADEICKRLA----------------PDSRLNPH-RSLLGTG-NYDVNVIMAALQGLGLAAVWWDRR-RPLSQLA
AT3   ----------MESIFHEKQEGSLCAQHCLNNLLQGEYFSPVELSSIAHQLDEEERMRMAEGGVTSEDYRTFLQQP-SGNMDDSGFFSIQVISNALKVWGLELILFNSPEYQRLRID
AT3L  ----------MDFIFHEKQEGFLCAQHCLNNLLQGEYFSPVELASIAHQLDEEERMRMAEGGVTSEEYLAFLQQP-SENMDDTGFFSIQVISNALKFWGLEIIHFNNPEYQKLGID


Jos2  GGG  EEEEEEE              EEEEEEEEE  EEEE        EEEE HHHHHHHHHHHHHH   EEEEEEEHHHHHH
AT3   GGG  EEEEEE                EEEEEEEE  EEEEEE      EEE H-HHHHHHHHHHHHH   EEEEEE                   HHHH
AT3L  GGG  EEEEEE                 EEEEEEE  EEEE        EEE H-HHHHHHHG-GGG   EEEEEEE                   HHHHHH
Jos2  LPQVLGLILNLPSPVSLGLLSLPLRRRHWVALRQVDGVYYNLDSKLRAPEALGDEDGVRAFLAAALAQGLCEVLLVVTKEVEEKGSWLRTD-
AT3   PINERSFICNYK--------------EHWFTVRKLGKQWFNLNSLLTGPELISD-TYLALFLAQLQQE-GYSIFVVKGD-------------LPDCEADQLLQMIR
AT3L  PINERSFICNYK--------------QHWFTIRKFGKHWFNLNSLLAGPELISD-TCLANFLA-RLQQQAYSVFVVKGD-------------LPDCEADQLLQI--
```

Figure S4. Secondary structure comparison between Josephin-2, ataxin-3, and AT3L. DSSP (Kabsch & Sander, 1983; Touw *et al*., 2015) was used to perform secondary structure analyses on Josephin-2 (this work), ataxin-3 (PDB code 1YZB), and AT3L (PBD code 3O65). The results of the DSSP analysis are shown above the aligned sequences. The nomenclature used follows the DSSP convention:

H	alpha helix
E	extended
G	3_{10} helix

References

Kabsch, W., 1976. A solution for the best rotation to relate two sets of vectors. Acta Crystallogr. A32, 922-923.

Kabsch, W. & Sander, C., 1983. Dictionary of protein secondary structure: pattern recognition of hydrogen-bonded and geometrical features. Biopolymers 22, 2577-2637.

Touw, W. G., Baakman, C., Black, J., te Beek, T. A. H., Krieger, E., Joosten, R. P., Vriend, G., 2015. A series of PDB-related databases for everyday needs. Nucleic Acids Res. 43, D364-D368.
